# Nanofibrillated Cellulose and Copper Nanoparticles Embedded in Polyvinyl Alcohol Films for Antimicrobial Applications

**DOI:** 10.1155/2015/456834

**Published:** 2015-06-02

**Authors:** Tuhua Zhong, Gloria S. Oporto, Jacek Jaczynski, Changle Jiang

**Affiliations:** ^1^Division of Forestry and Natural Resources, West Virginia University, Morgantown, WV 26506, USA; ^2^Division of Animal and Nutritional Sciences, West Virginia University, Morgantown, WV 26506, USA

## Abstract

Our long-term goal is to develop a hybrid cellulose-copper nanoparticle material as a functional nanofiller to be incorporated in thermoplastic resins for efficiently improving their antimicrobial properties. In this study, copper nanoparticles were first synthesized through chemical reduction of cupric ions on TEMPO nanofibrillated cellulose (TNFC) template using borohydride as a copper reducing agent. The resulting hybrid material was embedded into a polyvinyl alcohol (PVA) matrix using a solvent casting method. The morphology of TNFC-copper nanoparticles was analyzed by transmission electron microscopy (TEM); spherical copper nanoparticles with average size of 9.2 ± 2.0 nm were determined. Thermogravimetric analysis and antimicrobial performance of the films were evaluated. Slight variations in thermal properties between the nanocomposite films and PVA resin were observed. Antimicrobial analysis demonstrated that one-week exposure of nonpathogenic *Escherichia coli* DH5*α* to the nanocomposite films results in up to 5-log microbial reduction.

## 1. Introduction

Currently in the Appalachian region there is a vast amount of low-value, low-quality hardwood that can potentially be used as feedstock for novel bioproducts. Only West Virginia generates 2.41 million dry tones of underutilized wood per year that might be a great source for nanocellulose production. Today the technology to separate and obtain wood polymers at nanoscale exists and it has been demonstrated with success; however, specific applications for these novel raw materials are still a challenge. Based on our preliminary results [1], one interesting application is the utilization of micro- and nanostructures of cellulose as templates and stabilizers for biocide nanoparticles with emphasis of application as antimicrobial nanocomposites in the packaging and/or medical industry.

Metals, such as copper and silver, are relatively common antimicrobial materials that can be incorporated as nanomaterials in thermoplastic films for packaging and/or medical industry [2–4]; however, to avoid leaching, to improve metal dispersion, and to improve the contact between the metal and the bacterial wall a supportive material might be required. In addition, it is expected that the metal ions can be released from the film in a controlled way to effectively prevent microbial growth. To date, the development of nanocomposite films fabricated from thermoplastic resins with cellulose nanofibers has emerged as a potentially effective approach for improving mechanical properties of these films [5, 6]. Our own preliminary findings provide evidence that these cellulose nanofibers could be used as support materials for copper nanoparticles improving also the antimicrobial properties of the films.

In general, the prevailing concept of grafting metal on the surface of cellulose derivatives involves trapping the metallic cations via electrostatic interactions with negatively charged groups (e.g., carboxylate and hydroxyl) present in the correspondent template. The presence of carboxyl groups on the cellulose backbone will help to stabilize and reduce copper ions on the cellulosic structure. In this research our focus was centered in the utilization of TEMPO-oxidized cellulose nanofibers as nanosized cellulose for the synthesis of copper nanoparticles. The hybrid material was embedded in a biodegradable polymer, polyvinyl alcohol, and the performance of the final film was evaluated.

TEMPO or 2,2,6,6-tetramethylpiperidine-1-oxyl molecule is a highly stable nitroxyl radical which is used extensively in the selective oxidation of primary alcohols to corresponding aldehydes and carboxylic acids [7]. In aqueous environments, TEMPO catalyzes the conversion of carbohydrate primary alcohols to carboxylate (COO-) functionalities in the presence of a primary oxidizing agent, for example, sodium hypochlorite (NaOCl). Various TEMPO-mediated oxidation reactions of mono-, oligo-, and polysaccharides for regioselective conversion of primary hydroxyls to carboxylate groups have been published elsewhere [8, 9]. In particular, wood celluloses can be converted to individual nanofibers 3-4 nm wide with several microns length by TEMPO-mediated oxidation and successive mild disintegration in water [7, 10, 11]. During this reaction significant amounts of C6 carboxylate groups are selectively formed on each cellulose microfibril surface without any changes to the original crystallinity or crystal width of wood celluloses.

Polyvinyl alcohol (PVA) is a commercial important water-soluble, semicrystalline, transparent, biocompatible, and biodegradable polymer. It has been successfully blended with several natural materials such as fibers and fillers for PVA mechanical properties improvements. With increasing interest in the use of biodegradable and sustainable plastics, PVA has been used in several applications such as tissue scaffolding, filtration materials, and membranes and drug delivery [6, 12, 13].

As mentioned previously, in this preliminary work a simple method was developed to produce hybrids of TEMPO nanofibrillated cellulose (TNFC) and copper nanoparticles. The hybrid material was subsequently embedded in polyvinyl alcohol thermoplastic resin and the final films were produced using a solvent casting method. The films were evaluated in terms of its morphology and thermal and antimicrobial properties.

## 2. Materials and Methods

### 2.1. Materials

TEMPO nanofibrillated cellulose (TNFC) (0.96 wt.%) from the Forest Product Laboratory, Madison, WI; technical-crystal cupric sulfate pentahydrate (CuSO_4_·5H_2_O) from Fisher Scientific, USA; sodium borohydride (NaBH_4_) (0.5 M) from Acros Organics, USA; poly(vinyl alcohol) (99-100% hydrolyzed, approx. M.W. 86000) was from Acros Organics, USA; Nonpathogenic* E. coli* DH5*α* (*α* substrain of DH5 described by Hanahan in 1985, “DH” stands for Douglas Hanahan) is a very sensitive microorganism. This* E. coli* contains mutations of the* recA* and* gyrA* (gyrase subunit A) genes that are necessary for DNA repair and replication. Therefore,* recA* and* gyrA* mutants have impaired ability to repair and recombine their DNA strands making the mutants sensitive to any stress including chemicals. This is why this sensitive and nonpathogenic* E. coli* was selected as a model microbial target in this study. Sterile trypticase soy broth (TSB) from Becton Dickinson, USA; Petrifilm* E. coli*/Coliform Count Plate from 3M, USA; Butterfield phosphate buffer from Hardy Diagnostics, USA.

### 2.2. Preparation of the Hybrid TNFC-Copper Nanoparticles

Hybrid TNFC-copper nanoparticles were prepared by introducing copper nanoparticles on TNFC substrate by the chemical reduction of cupric ions. Twelve grams of TNFC gel containing 0.96 wt.% cellulose nanofibers was dissolved in the deionized water under vigorous magnetic stirring. A predefined amount of CuSO_4_ solution (0.1 mol/L) as shown in Table 1 was added by drops into the TEMPO-oxidized cellulose nanofibers solution. The mixture of TNFC and cupric sulfate was subjected to high-speed mixing while the cupric sulfate solution was added. Then the mixture was allowed to react at room temperature for 3 h. After that, cupric ions were reduced to metallic copper or copper oxide nanoparticles by adding the predefined amount of reducing agent sodium borohydride (0.5 mol/L).

### 2.3. Preparation of PVA/TNFC and PVA/TNFC-Copper Nanoparticles Nanocomposite Films

Polyvinyl alcohol/TNFC-copper nanoparticle nanocomposite films were prepared by solvent casting method described elsewhere [14]. Ninety milliliters of deionized water was heated to 90°C using a hot plate. Upon the desired temperature, 10 g of PVA was sprinkled into the hot water under vigorous magnetic stirring, after all PVA was added; the beaker will be covered; the mixture was heated at 90°C for 2 h. Subsequently, hybrid TNFC-copper nanoparticles solution was added by drops into clear PVA solution under vigorous magnetic stirring for 2 h. The resulting solution was transferred to glass dish and put in the desiccator to degas for 24 h under vacuum and then put in the oven at 50°C for 24 h. PVA films with different copper concentration were formed and the compositions of the film were shown in Table 2. According to the weight ratio of copper in the composites, that is, 0.4, 0.5, and 0.6 wt.%, the nanocomposite films were coded as PVA/TNFC-Cu0.4, PVA/TNFC-Cu0.5, and PVA/TNFC-Cu0.6. Pure PVA and PVA/TNFC films were also prepared as control for antimicrobial testing.

### 2.4. Transmission Electron Microscopy (TEM)

The morphology and particle size of copper nanoparticles on TNFC substrate were observed by JEOL TEM-2100 instrument (Tokyo, Japan) operating at 120 kV. TEM samples were typically prepared by dropping the hybrid TNFC-copper nanoparticles solution on a 200-mesh Nickel grid coated with a carbon film.

### 2.5. Thermogravimetric Analyzer (TGA) Characterization

The thermal behaviors of pure PVA and its nanocomposite films were determined by TA Q50 thermogravimetric analyzer (Delaware, USA), with temperature ramp-up rate of 10°C/min while being purged with nitrogen at a flow rate of 20 mL/min. The sample weight was chosen between 3 mg and 4 mg for all of the samples tested.

### 2.6. Determination of Antimicrobial Activity of PVA/TNFC-Copper Nanoparticles Nanocomposite Films

*E. coli* lyfo-disks were reconstituted by crushing one pellet using a sterile spatula in 0.5 mL of sterile TSB. The content was aseptically transferred to 99.5 mL of sterile TSB and allowed to grow aerobically at 37°C for 24 h in an incubator/shaker set at 150 rpm (C24, New Brunswick Scientific, New Jersey, USA). This procedure yielded 100 mL of* E. coli* stock culture. For experiments, a loopful of the stock culture was transferred to 100 mL of sterile TSB followed by incubation at 37°C for 24 h in the C24 incubator/shaker set at 150 rpm. This procedure yielded a culture with appropriately 10^8^ colony-forming units per milliliter (CFU/mL). A 2 mL aliquot of such culture was transferred to a surface of pure PVA, PVA/TNFC, and PVA/TNFC-copper nanoparticles films and incubated at room temperature for 1 week. To prevent excessive evaporation of the* E. coli* culture from the surface of films, the films were kept in an aerobic environment with saturated humidity. After 1-week exposure, 1 mL of* E. coli* culture was removed from the surface of films to enumerate* E. coli* survivors. Prior to removal, the* E. coli* culture on films was carefully mixed to obtain equal cell distribution. Enumeration was performed by a standard serial 10-fold dilution procedure and spread plating in a biosafety cabinet under aseptic procedures [15, 16]. A 1 mL aliquot of the* E. coli* culture removed from the surface of films was aseptically mixed with 9 mL of diluent (Butterfield phosphate buffer, Hardy Diagnostics, Santa Maria, CA, USA) followed by shaking the diluent bottle to uniformly distribute bacterial cells. Subsequent serial 10-fold dilutions were aseptically made by taking 10 mL of diluted sample and transferring it to a 90 mL diluent bottle. Survivors were enumerated on selective medium (Petrifilm* E. coli*/Coliform Count Plate, 3M, St. Paul, MN, USA) using a standard spread-plating technique. A 1.0 mL aliquot of each serial 10-fold dilution was pipetted and spread on 3M Petrifilm plates. The 3M Petrifilm plates were incubated at 35°C for 48 h [17]. Only plates with 15–150 colonies were counted.

Experiments were independently triplicated (*n* = 3). Enumeration of* E. coli* survivors in each equipment was performed in duplicate. Mean values for* E. coli* survivors were used to calculate log reductions of* E. coli* on the tested films (Figure 5). Differences between treatments (i.e., different films) were tested using the Least Significant Difference (LSD) test. All statistical analyses of data were performed using JMP version 12 Statistical Software (Statistical Discovery, from SAS).

## 3. Results and Discussion

### 3.1. Morphology of Copper Nanoparticles on TNFC Template

**Figure 1:**
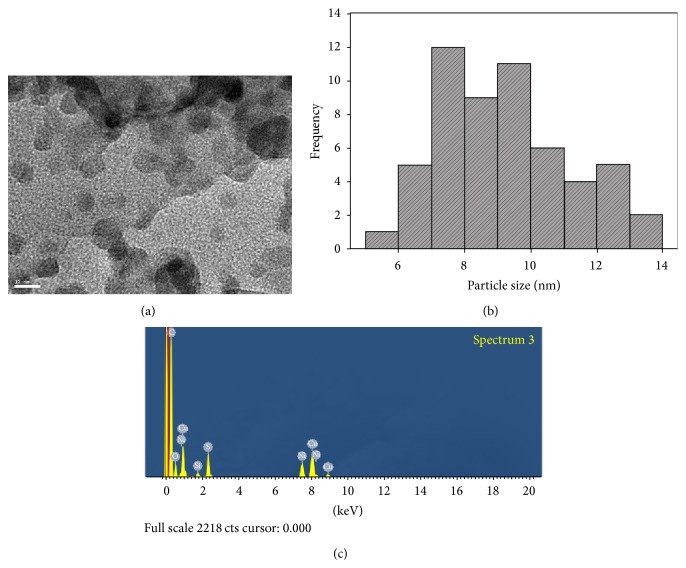
(a) TEM images of hybrid TNFC-copper nanoparticles (scale bar = 10 nm); (b) histogram of particle size distribution (*n* = 55 particles); (c) EDX spectrum of the hybrid TNFC-copper nanoparticles.

Figure 1 displays the TEM image of hybrid TNFC-copper nanoparticles (a), particle size histogram (b), and its corresponding EDX spectrum (c). Spherical copper nanoparticles with the particle size ranging from 5 nm to 14 nm and with average particles size 9.2 ± 2.0 nm are observed. EDX confirms the formation of copper nanoparticles by exhibiting peaks at approximately 8 keV (Figure 1(c)).

### 3.2. TGA Analysis

**Figure 2:**
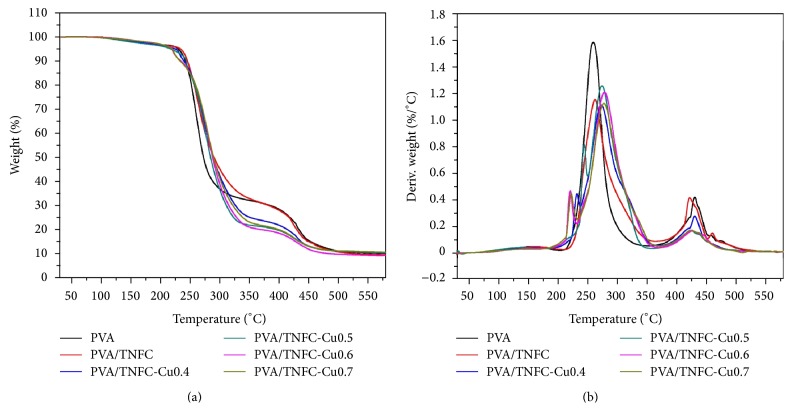
(a) TGA curves for the pure PVA, PVA/TNFC film, and PVA/TNFC-Cu composite films; (b) the corresponding DTG curves. Peak temperatures (*T*_max⁡_) of PVA, PVA/TNFC, PVA/TNFC-Cu0.4, PVA/TNFC-Cu0.5, PVA/TNFC-Cu0.6, and PVA/TNFC-Cu0.7 were 260, 263, 274, 275, 278, and 278°C, resp.).

Figure 2 shows typical TGA and DTG curves of the PVA composite films. All the PVA/TNFC-Cu composite films exhibited four distinct weight loss stages at 30–210°C (loss of weakly physic-sorbed water), 210–230°C (decomposition of TNFC-copper nanoparticles nanocomposites, the thermal behavior of TNFC-copper nanoparticles is similar to that of carboxymethyl cellulose-copper nanoparticles reported by Nadagouda and Varma [18]), 230–380°C (decomposition of side chain of PVA), and 380–550°C (decomposition of main chain of PVA). Major weight losses were observed in the range of 210–550°C, which corresponded to the structural decomposition of PVA and thermal degradation of TNFC. *T*_max⁡_ is the decomposition temperature corresponding to the maximum weight loss and relates to the maximum decomposition rate. In Figure 2(b), we can see that *T*_max⁡_ of PVA/TNFC-Cu composite films shifted to higher temperature compared to that of pure PVA and PVA/TNFC; an increase of *T*_max⁡_ was observed from 260 to 278°C for PVA and PVA/TNFC-Cu0.7 composite film, respectively. The thermal decomposition of PVA/TNFC-Cu films shifted slightly toward high temperature, suggesting that the composite films had higher thermal stability, which can be attributed to the presence of copper nanoparticles embedded in the PVA matrix.

### 3.3. Antimicrobial Activity of PVA/TNFC-Copper Nanoparticles Films

**Figure 3:**
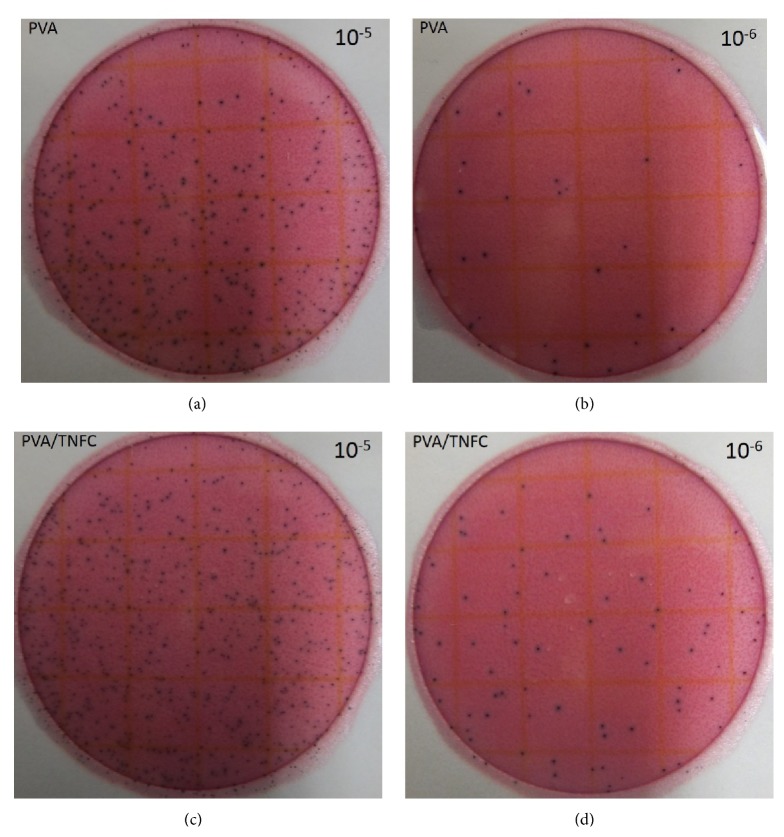
Bacterial enumeration of the 24 h* Escherichia coli* culture exposed to control materials: (a, b) PVA film and (c, d) PVA/TNFC film for 1 week at room temperature. (a, c) Representative Petrifilm plates for 10^−5^ dilution; (b, d) representative Petrifilm count plates for 10^−6^ dilution.

**Figure 4:**
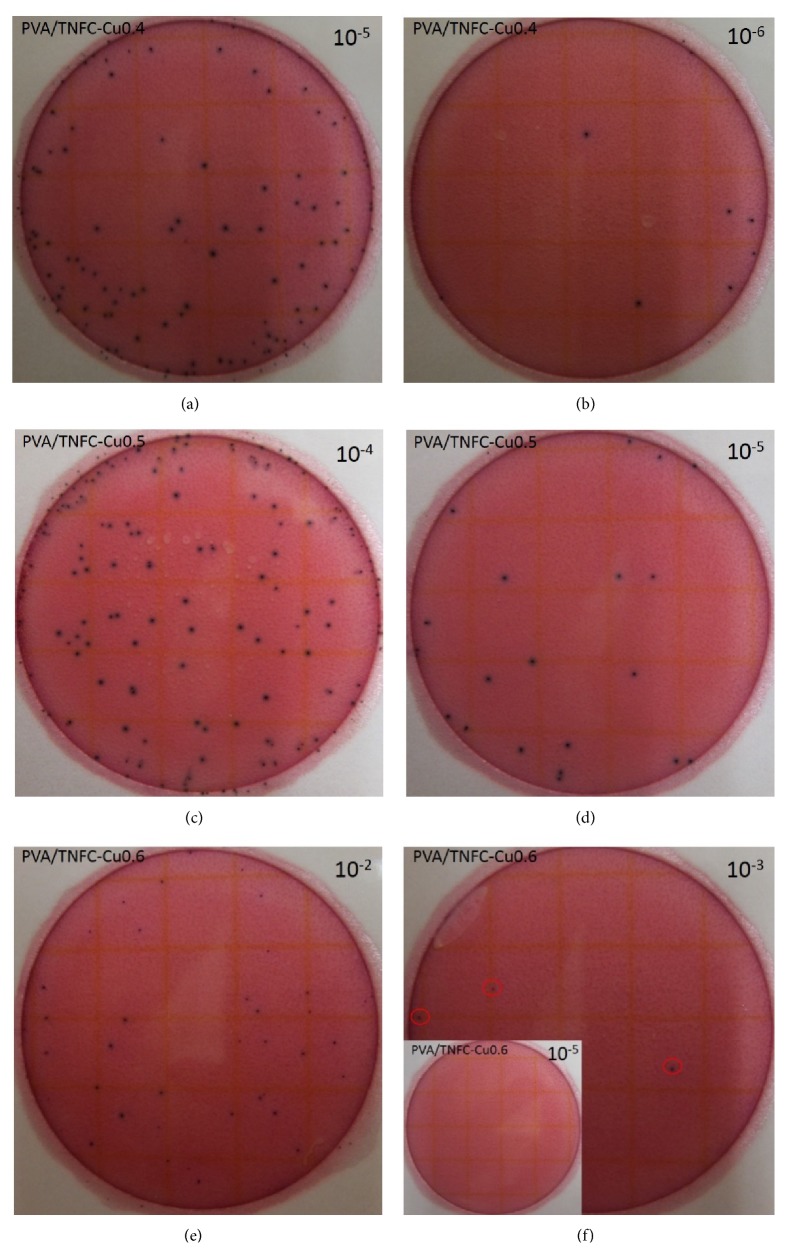
Bacterial enumeration of the 24 h* Escherichia coli* culture exposed to PVA nanocomposite film containing various copper content: (a, b) PVA/TNFC-Cu0.4, (c, d) PVA/TNFC-Cu0.5, and (e, f) PVA/TNFC-Cu0.6 films for 1 week at room temperature. (a–f) Representative Petrifilm count plates for various dilutions.

The antimicrobial properties of pure PVA, PVA/TNFC films, and PVA/TNFC-copper nanoparticle films with different copper concentration were tested against* E. coli*. Figures 3 and 4 show representative images of visual examples of different serial 10-fold dilutions for* E. coli* survivors following their exposure to pure PVA and PVA/TNFC films (Figure 3) and PVA/TNFC-copper nanoparticle films with different copper concentration (Figure 4). The initial concentration of* E. coli* (i.e., prior to exposure to films) was approximately 10^8^ CFU/mL. Figure 3 shows relatively minimal reduction of the initial* E. coli* concentration following exposure to pure PVA and PVA/TNFC films, while Figure 4 shows a trend of increasing microbial reduction as a function of greater copper nanoparticles concentration in films.

**Figure 5:**
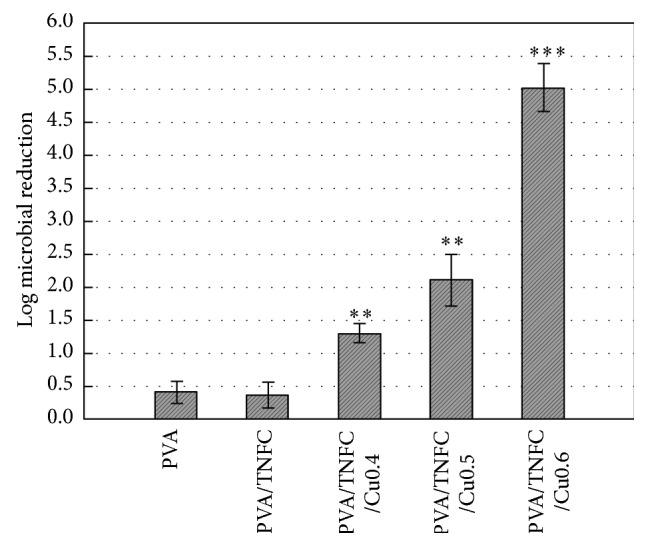
Microbial reduction induced by PVA/TNFC-copper nanoparticles after 1-week exposure. The asterisks refer to significant levels compared to one of the controls: PVA/TNFC, *P* < 0.05 (∗), *P* < 0.01 (∗∗), and *P* < 0.001 (∗∗∗).

The counts from enumeration of* E. coli* survivors (representative images shown in Figures 3 and 4) were log-converted and used to determine* E. coli* reductions as a function of exposure to different films (Figure 5). Figure 5 shows that PVA and PVA/TNFC films resulted in similarly (*P* > 0.05) minimal reduction of* E. coli*. However, increasing concentration of copper nanoparticles in PVA/TNFC films resulted in greater (*P* < 0.05) reduction of* E. coli*. After 1-week exposure of* E. coli* to films containing 0.4%, 0.5%, and 0.6% of copper nanoparticles, reduction of* E. coli* has gradually increased (*P* < 0.05) and reached about 5-log reduction for the highest inclusion of copper nanoparticles in the film. Based on Figure 5, embedding copper nanoparticles in the PVA/TNFC film results in inactivation of* E. coli* that shows greater efficacy with higher concentration of copper nanoparticles. However, it needs to be emphasized that* E. coli* used in this study as a microbial target was a very sensitive strain; and, therefore, the antimicrobial efficacy of copper nanoparticles would likely be less profound for a typical foodborne pathogen such as* E. coli* O157:H7. Further research using more resistant foodborne pathogens is recommended.

Even though the antimicrobial mechanism of copper nanoparticles against microorganisms has not been fully understood, three hypothetical mechanisms are the most widely accepted and reported in the literature: (1) copper nanoparticles accumulate in the bacterial membrane and cause changes in membrane permeability [19]; (2) reactive oxygen species (ROS) produced through Fenton-type reactions lead to free-radical-mediated cellular damage [20, 21]; (3) the release of copper ions from nanoparticles causes inactivation of enzymes and depletion of intracellular ATP as well as disruption of DNA replication [22].

In this study we propose that the antimicrobial effect of PVA/TNFC-copper nanoparticles films is directly related to the transfer of copper ions leaching in a controlled manner from the PVA matrix to bacterial cells. This is consistent with the traditional hypothesis that metal ions attach to the negatively charged bacterial cell wall, resulting in disruption of cell wall permeability and thus inducing protein denaturation and finally cell death. PVA is a hydrophilic polymer; and, therefore, it is hygroscopic. Water sorption may induce the release of copper ions trapped in nanoparticles within the PVA matrix because of surface oxidation that occurs when copper nanoparticles are exposed to oxygen [4].

## 4. Conclusions

Copper nanoparticles with average diameter of 9.2 ± 2.0 nm were successfully synthesized on the TEMPO nanofibrillated cellulose. The decomposition temperature, corresponding to the maximum weight loss, increased from 260°C for pure PVA, to 278°C for the PVA composite. The incorporation of hybrid TNFC-copper nanoparticles within PAV matrix endows the resulting composite films with antimicrobial properties. The PVA film containing copper content up to 0.6 wt.% exhibited a strong antimicrobial activity against* E. coli *DH*5α*, resulting in up to 5-log microbial reduction. The results suggest that TNFC-copper nanoparticles nanocomposites as antimicrobial nanofillers are valuable for PVA applications.

## Figures and Tables

**Table 1:** Preparation of the hybrid TNFC-copper nanoparticles.

Sample	TNFC gel (g)	TNFC (g)	CuSO_4_ (0.1 mol/L) (mL)	NaBH_4_ (0.5 mol/L) (mL)	Total liquid amount (mL)
TNFC-copper nanoparticles (1)	12	0.1152	6.4	2.56	30
TNFC-copper nanoparticles (2)	12	0.1152	8.0	3.2	30
TNFC-copper nanoparticles (3)	12	0.1152	9.6	3.84	30
TNFC-copper nanoparticles (4)	12	0.1152	11.2	4.42	30

**Table 2:** Preparation of the PVA/TNFC-copper nanoparticles nanocomposite films.

Composition	Sample code			
	PVA/TNFC-Cu0.4	PVA/TNFC-Cu0.5	PVA/TNFC-Cu0.6	PVA/TNFC-Cu0.7
PVA (g)	10	10	10	10
TNFC-copper nanoparticles (1) (mL)	30	—	—	—
TNFC-copper nanoparticles (2) (mL)	—	30	—	—
TNFC-copper nanoparticles (3) (mL)	—	—	30	—
TNFC-copper nanoparticles (4) (mL)	—	—	—	30
Copper content within final films (wt. %)	0.4	0.5	0.6	0.7
